# Etiological explanation, treatability and preventability of childhood autism: a survey of Nigerian healthcare workers' opinion

**DOI:** 10.1186/1744-859X-8-6

**Published:** 2009-02-12

**Authors:** Muideen Owolabi Bakare, Ahamefule O Agomoh, Peter O Ebigbo, Julian Eaton, Kevin O Okonkwo, Jojo U Onwukwe, Gabriel M Onyeama

**Affiliations:** 1 Child and Adolescent Unit, Federal Neuro-Psychiatric Hospital, New Haven, Enugu, Enugu State, Nigeria; 2 General/Forensic Unit, Federal Neuro-Psychiatric Hospital, New Haven, Enugu, Enugu State, Nigeria; 3 Department of Psychological Medicine, University of Nigeria Teaching Hospital, (UNTH), Enugu, Enugu State, Nigeria; 4 West Africa CBM National Co-ordination Office, P.O. Box 8451, Wuse, Abuja, Nigeria; 5 Community Psychiatry Unit, Federal Neuro-Psychiatric Hospital, New Haven, Enugu, Enugu State, Nigeria

## Abstract

**Background:**

Because of their peculiar sociocultural background, healthcare workers in sub-Saharan African subcultures may have various conceptions on different aspects of autism spectrum disorders (ASD), such as etiology, treatment and issues of prognosis. These various conceptions, if different from current knowledge in literature about ASD, may negatively influence help-seeking behavior of parents of children with ASD who seek advice and information from the healthcare workers. This study assessed the opinions of healthcare workers in Nigeria on aspects of etiology, treatability and preventability of childhood autism, and relates their opinions to the sociodemographic variables.

**Methods:**

Healthcare workers working in four tertiary healthcare facilities located in the south-east and south-south regions of Nigeria were interviewed with a sociodemographic questionnaire, personal opinion on etiology, treatability and preventability of childhood autism (POETPCA) questionnaire and knowledge about childhood autism among health workers (KCAHW) questionnaire to assess their knowledge and opinions on various aspects of childhood autism.

**Results:**

A total of 134 healthcare workers participated in the study. In all, 78 (58.2%), 19 (14.2%) and 36 (26.9%) of the healthcare workers were of the opinion that the etiology of childhood autism can be explained by natural, preternatural and supernatural causes, respectively. One (0.7%) of the healthcare workers was unsure of the explanation of the etiology. Knowledge about childhood autism as measured by scores on the KCAHW questionnaire was the only factor significantly associated with the opinions of the healthcare workers on etiology of childhood autism. In all, 73 (54.5%) and 43 (32.1%), of the healthcare workers subscribed to the opinion that childhood autism is treatable and preventable respectively. Previous involvement with managing children with ASD significantly influenced the opinion of the healthcare workers in subscribing to treatability of childhood autism, while working experience of less than 6 years among the healthcare workers significantly influenced the opinion of the healthcare workers in admitting to believing in the preventability of childhood autism.

**Conclusion:**

In designing policies and programs to change negative opinions or beliefs of healthcare workers about childhood autism, there is a need for baseline information such as this survey. Changing the negative opinions or beliefs of the healthcare workers about childhood autism should encourage appropriate help-seeking behavior among parents of children with ASD who may be seeking advice or information from the healthcare workers. This would encourage early interventions, which are essential to prognosis of childhood autism.

## Background

Adequate and necessary dissemination of information to clients in a healthcare system is part of the essential ingredients of ensuring optimal healthcare performance [1,2]. Information that is provided by healthcare workers to clients can be grossly inadequate, and the content of such information is often influenced by the knowledge base of the healthcare workers and the healthcare workers' opinions on etiology and other issues relating to prognosis of a particular ailment. Observation of the influence of healthcare worker knowledge and opinions on quantity and quality of information provided to clients has particularly been made in cases of autism spectrum disorders (ASD) [3].

Help-seeking behavior and pathway to care are extremely influenced by the opinions or beliefs of the clients and caregivers on the etiology of a particular disorder. Help-seeking behavior and the pathway to care among clients utilizing mental healthcare systems has consistently been noted to be influenced by cultural perspectives and beliefs of clients and caregivers on the etiology of the particular mental disorder in question [4-6].

In the context of sub-Saharan Africa, where healthcare workers living among the general population in the community often play a pivotal role in offering medical advice and providing information on healthcare related issues to people in their immediate community, healthcare workers' cultural perspectives and their opinions or beliefs on etiology of developmental disorders such as ASD would greatly influence the help-seeking behavior of parents of children with ASD living around them in the immediate community.

Childhood autism is a developmental disorder that had been observed to have better prognosis with early intervention, which is often achieved through early recognition and diagnosis [7-9]. The nature of information provided by the healthcare workers to parents of children with childhood autism could influence help-seeking behavior and early intervention. It has been noted previously that the ability of healthcare workers to provide adequate and necessary information to parents of children with childhood autism is often an indicator of their knowledge, perception of the etiology and awareness of issues related to the prognosis of childhood autism [3].

Given the peculiar subcultures of sub-Saharan Africa including Nigeria, where knowledge and awareness about childhood autism is still relatively low and there exists an imperative need for education of healthcare workers and the public to raise levels of awareness, it is of paramount importance to have some insight into the baseline opinions of healthcare workers in this environment on etiology and issues relating to prognosis and preventability of childhood autism.

This study therefore assessed the baseline opinions of healthcare workers working in tertiary healthcare facilities in the south-east and south-south regions of Nigeria on the etiology, treatability and preventability of childhood autism. It also examined the associations between sociodemographic variables of the healthcare workers and their opinions on issues of etiology, treatability and preventability of childhood autism.

## Methods

### Locations

The locations of the study were four tertiary healthcare facilities located in the south-east and far south regions of Nigeria. Two of these tertiary healthcare facilities are specialized psychiatric hospitals, while the other two are the pediatric departments of two university teaching hospitals. The healthcare facilities where participants were interviewed were University of Calabar Teaching Hospital (UCTH) and Federal Psychiatric Hospital, Calabar, both of which are located in Cross River State (south-south region of Nigeria), and Ebonyi State University Teaching Hospital (EBSUTH), Abakaliki, Ebonyi State and Federal Neuro-Psychiatric Hospital, Enugu, Enugu State, both of which are located in the south-east region of Nigeria.

### Participants and sampling method

Participating healthcare workers were nurses, either working at the two specialized psychiatry facilities or working in department of pediatrics of the two university teaching hospitals involved in the study. The educational qualifications of the nurses were mostly diplomas in general and psychiatric nursing, and they had been working in their various areas of specialty for at least 1 year. The study was a point survey of opinions of healthcare workers on some aspects of ASD. A point-sampling method that involved all nurses in their duty posts in the four different institutions on the particular day the data were collected was employed. Therefore, all nurses in their duty posts in the four different institutions on that particular day were interviewed.

### Ethical considerations

Ethical approval for this study was obtained from the Institutional Review Board (IRB) of Federal Neuro-Psychiatric Hospital, New Haven, Enugu, Enugu State, Nigeria.

### Sociodemographic questionnaire

This was used to obtain sociodemographic information of the healthcare workers such as sex, age, marital status, and area of specialty, among others.

### Personal opinion on etiology, treatability and preventability of childhood autism (POETPCA) questionnaire (Appendix 1)

This questionnaire was designed to obtain information from healthcare workers about their opinion on etiology, treatability and preventability of childhood autism. The first part of the questionnaire dealt with opinions of the healthcare workers on the etiology of childhood autism. In this part of the questionnaire, healthcare workers were requested to make a choice from a list of four options as to their own opinion on what they thought could be the causal explanation of childhood autism. The four options were: natural causes, preternatural causes, supernatural causes and not sure. For each option they chose, they were further requested to explain or specify what they meant. The second part of the questionnaire dealt with the opinion of the healthcare workers on treatability and preventability of childhood autism. It contained the following questions:

• In your own opinion, do you think childhood autism can be treated?

• In your own opinion, do you think childhood autism can be prevented?

Both questions had the answer options of 'YES' or 'NO', and also further options or space for the healthcare workers to explain or state the reasons for their choice (see Appendix 1).

### Knowledge about childhood autism among health workers (KCAHW) questionnaire [10]

The KCAHW questionnaire measures knowledge about childhood autism aimed at early recognition and diagnosis of ASD among healthcare workers. It is a self-administered questionnaire that contains a total of 19 questions. Each of the questions has three options to choose from, with only one of these three options being correct. The correct option on each question attracts a score of 1, while the other two incorrect options each attract a score of 0. The questionnaire is further divided into four domains. Domain 1 assesses areas of impairment in social interaction and contains eight questions. Domain 2 addresses impairment in areas of communication and language development and contains one question. Domain 3 assesses areas of obsessive and compulsive patterns of behavior found in children with ASD and contains four questions. Domain 4 addresses information on what type of disorder childhood autism is, possible comorbid conditions, and onset of childhood autism in affected children, and contains six questions. Therefore, the KCAHW questionnaire contains a total of 19 questions and possible maximum and minimum total scores of 19 and 0, respectively, when the individual domain scores are added together. The content of the questionnaire and a reliability test for the questionnaire have been described in detail in a previous study [10]. This questionnaire was used to assess knowledge aimed at early recognition and diagnosis of ASD among the healthcare workers that participated in the study.

### Procedure

The three questionnaires were distributed to the participating healthcare workers to complete. It was ensured that the questionnaires were completed there and then and collected back immediately from the healthcare workers because they were meant for a point-of-time assessment of opinions on aspects of ASD and knowledge about childhood autism.

### Data analysis

The data were analyzed using SPSS v.15 (SPSS, Chicago, IL, USA). The chi square test was used to determine possible significant associations between sociodemographic variables and opinions of the healthcare workers on issues of etiology, treatability and preventability of childhood autism. The opinions of the healthcare workers on etiological explanation, treatability and preventability of childhood autism were also related with the mean score of the healthcare workers on the KCAHW questionnaire. p Values ≤ 0.05 were considered significant.

## Results

A total of 134 healthcare workers, which represented the total population on point sampling of the four different institutions studied, consented to participate in the study. There were 71 (53.0%) males and 63 (47.0%) females. The mean age of the participants was 35.89 ± 7.56 years. The mean score of participated healthcare workers on the KCAHW questionnaire was 12.35 ± 4.40. Other sociodemographic variables are shown in Table 1.

**Table 1:** Sociodemographic variables of the healthcare workers

**Sociodemographic variables**	**n (%)**
Age group (years):	
20 to 29	28 (20.9)
30 to 39	57 (42.5)
40 to 49	44 (32.8)
50 and above	5 (3.7)
Gender:	
Male	71 (53.0)
Female	63 (47.0)
Marital status:	
Never married	38 (28.4)
Married	91 (67.9)
Separated/divorced	1 (0.7)
Widowed	4 (3.0)
Area of specialty:	
Pediatrics	21 (15.7)
Psychiatry	113 (84.3)
Working experience (years):	
1 to 5	61 (45.5)
6 to 10	9 (6.7)
11 to 15	16 (11.9)
16 to 20	41 (30.6)
20 and above	7 (5.2)
Geographical region:	
South-east	62 (46.3)
South-south	72 (53.7)
Previous involvement with management of children with autism spectrum disorders (ASD):	
Previous involvement	65 (48.5)
No previous involvement	69 (51.5)
Scores on KCAHW questionnaire:	
KCAHW questionnaire score ≥ mean score	94 (70.1)
KCAHW questionnaire score < mean score	40 (29.9)

KCAHW, knowledge about childhood autism among health workers.

### Opinions of the healthcare workers on etiology of childhood autism

A total of 78 (58.2%) of the healthcare workers were of the opinion that the etiology of childhood autism is natural, while 36 (26.9%) and 19 (14.2%) subscribed to supernatural and preternatural causes, respectively. One healthcare worker (0.7%) was not sure of the etiology of childhood autism. Those healthcare workers that subscribed to natural causes were likely to attribute the etiology of childhood autism to genetics, birth injury, and maternal infections during pregnancy among other reasons. Those who subscribed to both preternatural and supernatural causes were likely to give explanations such as lineage curses, enemies, and action of the devil, among others.

### Association between opinions of healthcare workers on explanation of the etiology of childhood autism and sociodemographic variables of the healthcare workers

Opinions of the healthcare workers on etiology of childhood autism were only significantly associated with their scores on the KCAHW questionnaire. Healthcare workers having the mean score and above on the KCAHW questionnaire were more likely to subscribe to natural causes for childhood autism compared to those who had scores lower than the mean score on the KCAHW questionnaire (chi square = 10.6, degrees of freedom (df) = 3, p value = 0.014). Table 2 shows the association between the opinions of healthcare workers on explanation of the etiology of childhood autism and sociodemographic variables.

**Table 2:** Association between opinions of healthcare workers on explanation of the etiology of childhood autism and sociodemographic variables of the healthcare workers

**Sociodemographic variables**	**Level of association (p value)**
Age groups (years)	0.808
Gender	0.353
Marital status	0.732
Area of specialty	0.320
Working experience (years)	0.219
Geographical region	0.679
Previous involvement with management of children with autism spectrum disorders (ASD)	0.399
Mean score on KCAHW questionnaire	0.014^a^

^a^Significant association.

KCAHW, knowledge about childhood autism among health workers.

### Opinions of healthcare workers on the treatability and preventability of childhood autism

A total of 73 (54.5%) of the healthcare workers were of the opinion that childhood autism is treatable, while 43 (32.1%) of the healthcare workers were of the opinion that childhood autism is preventable. The healthcare workers that subscribed to the opinion that childhood autism is treatable cited special education and behavioral therapy among others as possible modalities of treatment. The healthcare workers who were of the opinion that childhood autism is preventable cited avoiding maternal infection during pregnancy, avoiding birth injury, pleasing the ancestral spirit and avoiding sins, among others, as modalities of prevention.

### Association between opinions of the healthcare workers on explanation of etiology, treatability and preventability of childhood autism

A significant association was found between the opinions of healthcare workers on explanation of the etiology and treatability of childhood autism, with those healthcare workers who subscribed to natural causes for childhood autism being more likely to have the opinion that childhood autism is treatable (chi square = 15.30, df = 3, p value = 0.002). There was also a significant association between the opinions of healthcare workers on explanation of the etiology and preventability of childhood autism, with those healthcare workers admitting to natural and preternatural causes more likely to subscribe to the opinion that childhood autism is preventable (chi square = 11.82, df = 3, p value = 0.008).

### Association between the opinions of healthcare workers on the treatability of childhood autism and sociodemographic variables of the healthcare workers

Opinions of healthcare workers on the treatability of childhood autism showed significant association with being previously involved with management of children with ASD, with those healthcare workers who have had previous involvement in managing children with ASD more likely to subscribe to the opinion that childhood autism is treatable when compared to those who had not been involved before in the management of children with ASD (chi square = 9.00, df = 1, p value = 0.003). Opinions of the healthcare workers on treatability of childhood autism showed near-significant association with geographical region, with the healthcare workers located in the south-east region of Nigeria more likely to express the opinion that childhood autism is treatable compared to those located in the south-south region of the country (chi square = 3.32, df = 1, p value = 0.068). Near-significant association was also found between opinions of the healthcare workers on treatability of childhood autism and their scores on the KCAHW questionnaire, with those healthcare workers having the mean score and above on the KCAHW questionnaire more likely to subscribe to the opinion that childhood autism is treatable when compared to those who had scores lower than the mean score on the KCAHW questionnaire (chi square = 3.30, df = 1, p value = 0.069). Table 3 shows the association between opinions of the healthcare workers on the treatability of childhood autism and sociodemographic variables of the healthcare workers.

**Table 3:** Association between opinions of healthcare workers on the treatability of childhood autism and sociodemographic variables of the healthcare workers

**Sociodemographic variables**	**Level of association (p value)**
Age groups (years)	0.503
Gender	0.420
Marital status	0.360
Area of specialty	0.493
Working experience (years)	0.663
Geographical region	0.068^a^
Previous involvement with management of children with autism spectrum disorders (ASD)	0.003^b^
Mean score on KCAHW questionnaire	0.069^a^

^a^Near-significant association; ^b^significant association.

KCAHW, knowledge about childhood autism among health workers.

### Association between the opinions of healthcare workers on the preventability of childhood autism and sociodemographic variables of the healthcare workers

A significant association was found between the opinions of healthcare workers on the preventability of childhood autism and years of working experience, with those healthcare workers with working experience below 6 years more likely to express the opinion that childhood autism is preventable (chi square = 12.92, df = 4, p value = 0.012). Near-significant association was found between opinions of the healthcare workers on preventability of childhood autism and gender, with females more likely to subscribe to the opinion that childhood autism is preventable compared to males (chi square = 3.15, df = 1, p value = 0.076). Near-significant association was also found between opinions of the healthcare workers on the preventability of childhood autism and age group, with those healthcare workers below 40 years of age more likely to express the opinion that childhood autism is preventable (chi square = 7.10, df = 3, p value = 0.069). Table 4 shows the association between the healthcare workers' opinions on preventability of childhood autism and their sociodemographic variables.

**Table 4:** Association between opinions of the healthcare workers on preventability of childhood autism and sociodemographic variables of the healthcare workers

**Sociodemographic variables**	**Level of Association (p value)**
Age groups (years)	0.069^a^
Gender	0.076^a^
Marital status	0.142
Area of specialty	0.895
Working experience (years)	0.012^b^
Geographical region	0.281
Previous involvement with management of children with autism spectrum disorders (ASD)	0.751
Mean score on KCAHW questionnaire	0.639

^a^Near-significant association; ^b^significant association.

KCAHW, knowledge about childhood autism among health workers.

## Discussion

Childhood autism is a pervasive developmental disorder, where a definitive etiology is still obscured. What is certain to date is that it occurs as a result of developmental problems in the brain that affect phenotypic areas of communication and social interaction, and it brings about restricted areas of interest or repertoire in the affected child [11]. A biologically determined etiology is therefore more likely in childhood autism. A little more than half of the healthcare workers that participated in this study subscribed to natural causes as explanation of the etiology for childhood autism. This is relatively encouraging, as the group of healthcare workers that were of this opinion are more likely to recommend orthodox practice as a source of help for parents of children with ASD, rather than alternative practices that may hinder early intervention.

Another interesting finding of this study was that the more apt the healthcare workers were at being able to recognize the symptoms of childhood autism (as reflected by their scores on the KCAHW questionnaire), the more likely they were to express an opinion of natural causes as explanation for the etiology of childhood autism. Promoting knowledge among healthcare workers about childhood autism through continuing education and special training would not only help in early recognition and diagnosis, but also positively influence the help-seeking behavior of parents of children with ASD who would come into contact with these healthcare workers.

Current knowledge advocates a multidisciplinary approach to the management of childhood autism. This management approach incorporates special education, behavioral therapy, social and communication skill training and psychotropic medications, if indicated [12]. With early intervention, positive changes have been observed overtime in symptom presentation among individuals with ASD [12]. Childhood autism can therefore be managed with reasonable improvement in symptoms presentation if recognized early. The principle behind the treatment of children with autism is the need for a multidisciplinary approach in management.

About 55% of the healthcare workers that participated in this study were of the opinion that childhood autism is treatable. This gave us some hope in the sense that these healthcare workers would be able to reassure parents of children with ASD and give appropriate information that would be necessary to their seeking help. However, what is lacking in most third-world regions such as Nigeria is a multidisciplinary approach to the management of children with developmental disabilities in general, largely due to the absence of trained professionals and lack of facilities for special needs of children with developmental disorders.

About 32% of the healthcare workers subscribed to the opinion that childhood autism is preventable. The premise for this position among the healthcare workers was often based on the theory of insult to the developing brain, either as a result of maternal infection, intrauterine infection or physical trauma to the brain from delivery or birth complications, and also the theory of supernatural causes as explanation for the etiology of childhood autism, which rest on (lack of) forgiveness of sins and trespasses, and cutting off links with cursed ancestral spirits among others. Current knowledge however has not afforded the scientific community the opportunity of knowing the definitive etiology of ASD and therefore definitive steps that are needed to ensure prevention also remained obscured.

Significant associations existed between the healthcare workers' opinions on the etiology, treatability and preventability of childhood autism. Essentially, the opinions of the healthcare workers that participated in the study on the etiology of childhood autism had significant influence on their opinions about whether childhood autism is treatable and/or preventable. This finding is in line with the general phenomenon that the etiology of any disorder often influences its treatment and preventability.

The opinions of the healthcare workers on whether childhood autism is treatable or can be managed were significantly influenced by previous involvement with managing children with ASD. Those healthcare workers who had been involved in the management of children with childhood autism were more likely to show optimism that the condition can be managed or treated.

The opinions of the healthcare workers on whether childhood autism is preventable were significantly influenced by number of years of working experience of the healthcare workers, with those who had less than 6 years work experience more likely to subscribe to the opinion that childhood autism is preventable. The reason for this finding is not clear, but it could be due to the limited experience of these healthcare workers in working with children with developmental disorders.

The significance of this baseline study is in assessing the areas where attention needs to be focused among healthcare workers in changing their negative attitudes and beliefs on various aspects of childhood autism; a process that is envisaged as necessary in promoting care for children with ASD and other developmental disorders in this environment. More attention therefore needs to be focused on specialized training on ASD for healthcare workers working with children and adolescents in this environment.

## Conclusion

The present information on the opinions of the participating healthcare workers on various aspects of childhood autism serve a baseline on which future policies and programs to change negative opinions and beliefs of healthcare workers in this environment on various aspects of childhood autism can be based.

Changing the negative opinions or beliefs of the healthcare workers about childhood autism would encourage appropriate help-seeking behavior among parents of children with ASD, who would be seeking advice or information from the healthcare workers. This would encourage early intervention, which is essential to the favorable prognosis of childhood autism.

## Competing interests

The authors declare that they have no competing interests.

## Authors' contributions

All authors contributed to the conception of the study. MOB was involved with writing the initial draft of the manuscript. MOB, AOA, POE, JE, KOO, JUO and GMO were involved in revising the manuscript. All authors read and approved the final draft of the manuscript.

## Appendix 1

### Personal opinion on etiology, treatability and preventability of childhood autism (POETPCA) questionnaire

Kindly answer the following questions to the best of your opinion. Please do not consult formal textbooks to answer these questions. Thank you for your time.

### Etiology of childhood autism

1.   In your own opinion, what is the likely causal explanation of childhood autism among the possible causal explanations listed below (tick one and give reasons for your choice)

a.   Natural causes (specify)...............................................................

b.   Preternatural causes (specify).........................................................

c.   Supernatural causes (specify).........................................................

d.   Not sure (specify).......................................................................

### Treatability and preventability of childhood autism

2.   In the questions below tick one appropriate option to the best of your opinion:

i.   In your own opinion, do you think childhood autism can be treated?

   (a) YES

   (b) NO (specify why).................................................................

ii.   In your own opinion, do you think childhood autism can be prevented?

   (a) YES

   (b) NO (specify why)...................................................................

## References

[B1] World Health Organization (WHO) (2000). How well do health systems perform?. World Health Report 2000.

[B2] Bramesfeld A, Wedegartner F, Elgeti H, Bisson S (2007). How does mental health care perform in respect to service users' expectations? Evaluating inpatient and outpatient care in Germany with the WHO responsiveness concept. BMC Health Serv Res.

[B3] Rhoades RA, Scarpa A, Salley B (2007). The importance of physician knowledge of autism spectrum disorder: results of a parent survey. BMC Pediatr.

[B4] Sheikh S, Furnham A (2000). A cross-cultural study of mental health beliefs and attitudes towards seeking professional help. Soc Psychiatry Psychiatr Epidemiol.

[B5] Cauce AM, Domenech-Rodriguez M, Paradise M, Cochran BN, Shea JM, Srebnik D, Baydar N (2002). Cultural and contextual influences in mental health help seeking: a focus on ethnic minority youth. J Consult Clin Psychol.

[B6] Bakare MO Psychological disorders in Nigerian children and adolescents, and their peculiarities. http://iacapap.ki.se/bulletins/Melbourne%20Supplement%202007.pdf.

[B7] Filipek PA, Accardo PJ, Baranek GT, Cook EH, Dawson G, Gordon B, Gravel JS, Johnson CP, Kallen RJ, Levy SE, Minshew NJ, Ozonoff S, Prizant BM, Rapin I, Rogers SJ, Stone WL, Teplin S, Tuchman RF, Volkmar FR (1999). The screening and diagnosis of autistic spectrum disorders. J Autism Dev Disord.

[B8] Gray KM (2001). Are there early features of autism in infants and pre-school children?. J Paediatr Child Health.

[B9] American Academy of Pediatrics, Committee on Children with Disabilities (2001). The pediatrician's role in the diagnosis and management of autistic spectrum disorders in children. Pediatrics.

[B10] Bakare MO, Ebigbo PO, Agomoh AO, Menkiti NC (2008). Knowledge about childhood autism among health workers (KCAHW) questionnaire: description, reliability and internal consistency. Clin Pract Epidemiol Mental Health.

[B11] Volkmar FR, Klin A, Sadock BJ, Sadock VA (2000). Pervasive developmental disorders. Kaplan and Sadock's Comprehensive Textbook of Psychiatry.

[B12] Holt G, Bouras N (2002). Autism and Related Disorders – The Basic Handbook for Mental Health, Primary Care and Other Professionals.

